# Multidrug-Resistant Bacteria Associated with Cell Phones of Healthcare Professionals in Selected Hospitals in Saudi Arabia

**DOI:** 10.1155/2018/6598918

**Published:** 2018-12-24

**Authors:** Saeed Banawas, Ahmed Abdel-Hadi, Mohammed Alaidarous, Bader Alshehri, Abdul Aziz Bin Dukhyil, Mohammed Alsaweed, Mohamed Aboamer

**Affiliations:** ^1^Department of Medical Laboratory Sciences, College of Applied Medical Sciences, Majmaah University, Majmaah 11952, Saudi Arabia; ^2^Department of Botany and Microbiology, Faculty of Science, Al-Azhar University, Assuit Branch, Cairo, Egypt; ^3^Department of Medical Equipment Technology, College of Applied Medical Sciences, Majmaah University, Majmaah 11952, Saudi Arabia

## Abstract

Cell phones may be an ideal habitat for colonization by bacterial pathogens, especially in hot climates, and may be a reservoir or vehicle in transmitting nosocomial infections. We investigated bacterial contamination on cell phones of healthcare workers in three hospitals in Saudi Arabia and determined antibacterial resistance of selected bacteria. A questionnaire was submitted to 285 healthcare workers in three hospitals, and information was collected on cell phone usage at the work area and in the toilet, cell phone cleaning and sharing, and awareness of cell phones being a source of infection. Screening on the Vitek 2 Compact system (bioMérieux Inc., USA) was done to characterize bacterial isolates. Of the 60 samples collected from three hospitals, 38 (63.3%) were positive with 38 bacterial isolates (4 Gram-negative and 34 Gram-positive bacteria). We found 38.3% of cell phones were contaminated with coagulase-negative staphylococci, particularly *Staphylococcus epidermidis* (10 isolates). Other bacterial agents identified were *S. aureus*, *S. hominis*, *Alloiococcus otitis*, *Vibrio fluvialis*, and *Pseudomonas stutzeri*. Antimicrobial susceptibility testing showed that most coagulase-negative staphylococci were resistant to benzylpenicillin, erythromycin, and rifampicin. Eight isolates were resistant to oxacillin, specifically *S. epidermidis* (3), *S. hominis* (2), and *S. warneri* (2). *A. otitis*, a cause of acute otitis media showed multidrug resistance. One isolate, a confirmed hetero-vancomycin intermediate-resistant *S. aureus*, was resistant to antibiotics, commonly used to treat skin infection. There was a significant correlation between the level of contamination and usage of cell phone at toilet and sharing. Our findings emphasize the importance of hygiene practices in cell phone usage among healthcare workers in preventing the transmission of multidrug-resistant microbes.

## 1. Introduction

The popularity of cell phones with healthcare professionals and lack of antiseptic practices make them potential routes of transmission of bacterial pathogens [1]. It has been reported that inanimate objects used by healthcare workers including cell phones act as important origins of nosocomial infections [2]. The cell phones of healthcare workers may act as reservoirs of nosocomial pathogens, which can be easily transmitted from the cell phone by the hands of a healthcare worker, thereby spreading bacterial isolates from one patient to another in various hospital wards [3]. Nosocomial infections are associated with significant morbidity and mortality. Studies have shown that the most common bacteria are coagulase-negative staphylococci, *Escherichia coli*, and *Pseudomonas* [4] Coagulase-negative staphylococci can invade the human body and cause serious infections, including hospital-acquired blood stream and skin infections [5]. The increasing significance of multidrug-resistant strains including staphylococci, among other etiologic agents of nosocomial infections, imposes on researchers the need to seek possible ways in the spread of these pathogens and ensure their robust and effective prevention. Therefore, the aim of the study was isolation and identification of bacteria from mobile phones. Moreover, we determined antibiotic resistance of the isolates.

## 2. Materials and Methods

### 2.1. Study Setting

This study was performed in three selected hospitals in Riyadh Province, Saudi Arabia. Sixty swab samples were collected from the cell phones of those volunteers who consented for two months between September and November 2017. Swab samples were collected by swabbing the top portion of the cell phones using the BD BBL™ culture swab™ collection and transport systems [6]. Aseptic practices were followed during the sampling process. Of the 60 samples collected, 23 samples were obtained from hospital A, 20 samples were from hospital B, and 17 samples were from hospital C. In addition, written informed consent was signed by all healthcare workers prior to sample collection. Deanship of Scientific Research at Majmaah University approved the study with approval ethical number (MUREC-Sept.25/COM-2017/120).

### 2.2. Bacterial Isolation and Characterization

Collected swab samples were inoculated on 5% sheep blood agar and MacConkey agar (Oxoid, UK) and incubated at 37°C for 48 hours. Different colonies were subcultured on nutrient agar and 5% sheep blood agar to get pure colonies of the isolates. The preliminary identification of all isolates was done using Gram stain and different biochemical tests including catalase, oxidase and coagulase tests [6].

### 2.3. Bacterial Identification

Identification of isolated bacteria at the species level was performed with the Vitek 2 Compact system (bioMérieux Inc., USA) according to the manufacturer's instruction. A bacterial suspension of each isolate was prepared by mixing the bacterial colony growing on blood agar with 0.45% saline sodium chloride solution to obtain a concentration of 0.5–0.63 McFarland units using the VITEK DensiCHEK™ colorimeter (bioMérieux). The suspensions (2 mL) were automatically loaded into the VITEK 2 ID system (bioMérieux), using GP ID REF21342 and GN ID REF21341 cards for the identification of Gram-positive and Gram-negative bacteria, respectively and the version 07.01 release software. The cards were read by kinetic fluorescence measurement, and the results reported within 3 h [7]. Quality control for Vitek was done using Gram-positive bacteria (*Enterococcus casseliflavus* ATCC 700327 and *Staphylococcus saprophyticus* BAA-750) and Gram-negative bacteria (*Enterobacter hormaechei* ATCC 700323 and *Stenotrophomonas maltophilia* ATCC 17666). Skim milk growth medium (20%) was used to store the identified isolates and frozen at −20°C [8].

### 2.4. Antimicrobial Susceptibility Testing

To determine antimicrobial susceptibility testing for the isolates, 145 *μ*L of the bacterial suspension was drawn into 3 mL of 0.45% saline solution to further adjust the bacterial cell density. Vitek cards were inoculated with the suspension vials and loaded into the Vitek 2 automated reader-incubator using AST-P580 (*S.* spp., *Enterococcus* spp., and *S. agalactiae*) and AST-N291 (Gram-negative bacilli) cards. Results were interpreted using Vitek 2 Compact software version 07.01 [7].

### 2.5. Questionnaire

We asked 285 healthcare workers in selected hospitals to complete a questionnaire, which included usage of cell phones at the work area and toilet, cleaning cell phones by disinfectants, and awareness that cell phones can serve as a source of infection.

### 2.6. Statistical Analysis

The correlation matrix by using Pearson's linear correlation coefficient [9] to discover the correlation between the contamination level and questionnaire variables (the usage of cell phones at the work area and toilet, cleaning cell phones by disinfectants, sharing, restriction of using cell phone at work, and awareness that cell phones can serve as a source of infection). The value of the correlation equal −1 indicates perfect negative correlation, and the value equal +1 indicates perfect positive correlation; *p* value < 0.05 was considered statistically significant.

## 3. Results and Discussion

### 3.1. Level of Contamination

**Figure 1:**
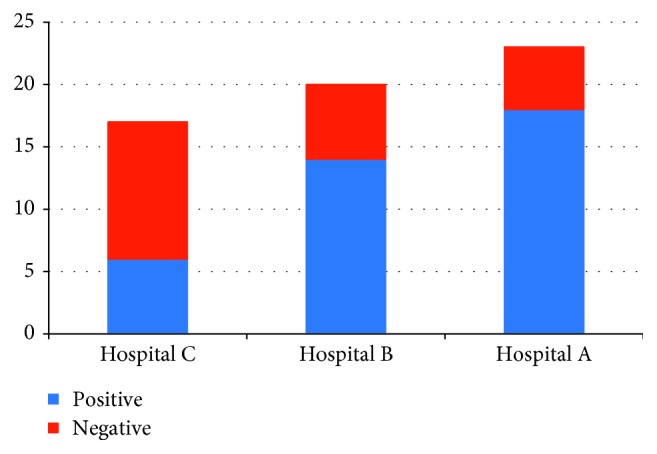
Bacterial frequency in collected samples from cell phones in selected hospitals.

The results showed that 38 (63.3%) of the 60 cell phone sample swabs collected from three hospitals were infected (Figure 1). Generally, the frequency of contaminated cell phones varied between the three selected hospitals, with the greatest contamination found in hospital A, where 18 (78.23%) of 23 samples were contaminated. Similarly, we found 70% (14/20) contamination in hospital B, while 35.39% (6/17) of cell phone sample swabs from hospital C were contaminated. Contamination of the healthcare environment coupled with nosocomial infections can lead to contamination of the cell phones of healthcare workers [10]. The hands of healthcare workers can be contaminated with different bacterial pathogens, and healthcare workers utilize cell phones in laboratories, hospital halls, operating rooms, and intensive care units [11]. Through every phone call, SMS, or other use, there is a risk that the cell phone comes into contact with contaminated areas of the human body by hand-to-hand contact or by hand to other areas, such as the mouth and ears [3]. Furthermore, cell phones may act as a favorable habitat for bacteria to colonize, especially under high temperature and humid conditions [12].

### 3.2. Bacterial Identification

Thirty-eight bacterial isolates belonging to coagulase-negative staphylococci (CNS) (60.5%), *Staphylococcus aureus* (2.6%), others Gram-positive (26.4%) including *Alloiococcus otitis*, *Micrococcus luteus, Globicatella sulfidifaciens*, *Kocuria rosea, Dermacoccus nishinomiyaensis* and *Facklamia hominis*), and Gram-negative bacteria (10.53%) including *Vibrio fluvialis*, *Alcaligenes faecalis*, *Acinetobacter lwoffii*, and *Pseudomonas stutzeri* were identified as cell phone contaminants. Eighteen isolates were isolated from hospital A and 14 isolates from hospital B, while only 6 isolates from hospital C. Samples from hospitals A and B had higher contamination rates than those from hospital C. In hospital A, 18 Gram-positive bacteria consisting of *S. hominis* subsp. *hominis* (18.4 %), *S. epidermidis* (18.4%), *S. capiti*s (2.6%), *Micrococcus luteus* (2.6%), *Globicatella sulfidifaciens* (2.6%), and *Facklamia hominis* (2.6%) were identified. In hospital B, 11 Gram-positive bacteria, specifically *S. epidermidis* (5.3 %), *S. lentus* (2.6%), *M. luteus* (5.3%), *Alloiococcus otitis* (5.3%), *Dermacoccus nishinomiyaensis* (5.3%), and *Kocuria rosea* (2.6%), and 4 Gram-negative bacteria, specifically *Vibrio fluvialis* (2.6%), *Alcaligenes faecalis* subsp. *faecalis* (2.6%)*, Acinetobacter lwoffii* (2.6%), and *Pseudomonas stutzeri* (2.6%), were identified. In hospital C, the 6 Gram-positive bacteria were identified as *S. aureus* (2.6%), *S. hominis* subsp. *hominis* (5.6%), *S. epidermidis* (2.6%), and *S. warneri* (5.3%) (Table 1).

Our study showed that coagulase-negative staphylococci were the most frequently isolated bacteria among healthcare workers (60.5%), particularly *S. epidermidis* and *S. hominis*. Our findings are similar to those of Zakai et al. [13] who reported coagulase-negative staphylococci were the most abundant isolates (68%) from contaminated cell phones of medical students in Saudi Arabia. It has been documented that handling contaminated inanimate objects during casual activities may cause hand-to-mouth transfer of pathogens. Furthermore, it has been predicted that cell phones can be an active origin of nosocomial infection as hand use to hold the phone comes in close contact with strongly contaminated body areas, such as the mouth, and ears [3]. In fact, nearly 30% of bacteria on cell phones are found on the hands of the owner [14]. Coagulase-negative staphylococci have the ability to create a biofilm on both animate and inanimate objects, which poses a particular threat for individuals receiving valve prostheses, implants, or catheters [15]. It was reported that coagulase-negative staphylococci are responsible for blood infections, of which *S. epidermidis* causes 67% of infections and other coagulase-negative staphylococci cause 33% [16].

### 3.3. Antimicrobial Susceptibility

Next, twenty-six Gram-positive bacteria were selected for antimicrobial susceptibility testing including 15 isolates from hospital A (*S. hominis* subsp. *hominis* (7), *S. epidermidis* (7), and *S. capiti*s (1)), 5 isolates from hospital B (*S. epidermidis* (2), *S. lentus* (1), and *A. otitis* (2)), and 6 isolates from hospital C (*S. aureus* (1), *S. hominis* subsp. *hominis* (2)*, S. epidermidis* (1), and *S. warneri* (2)).

As shown in Table 2, our antimicrobial susceptibility results indicate that most of the coagulase-negative isolates from the three hospitals were resistant to benzylpenicillin (MIC ≥ 0.5), erythromycin (MIC ≥ 8), and fusidic acid (MIC ≥ 32), with intermediate resistance to rifampicin (MIC ≤ 0.5). Resistance to oxacillin (MIC ≥ 4) was observed in *S. epidermidis* (30 %), *S. hominis* (22.2%), *S. warneri* (100%), and *S. lentus* (100%). Similarly, Asaad et al. [17] reported that coagulase-negative staphylococci isolates from nosocomial bloodstream infections in Najran (Saudi Arabia) were highly resistant to penicillin, oxacillin, and erythromycin, exhibiting sensitivity to vancomycin and teicoplanin. It has been believed that coagulase-negative staphylococci are important reservoirs of antimicrobial resistance genes and resistance-associated mobile genetic elements, which can be transferred between staphylococcal species. *S. hominis*, *S. epidermidis*, and *S. haemolyticus* are reported to be multiple drug resistant coagulase-negative staphylococci [18, 19]. It was demonstrated that mecA gene is transferred from coagulase-negative staphylococcal species to *S. aureus* in vivo and has a role in emergence of more successful *S. aureus* clones, cell adherence, and invasion [20, 21].

Interestingly, one isolate was confirmed as hetero-vancomycin intermediate-resistant *S. aureus* (hVISA) by standard Etest methods [22]. It was resistant to antibiotics commonly used to treat skin infection including benzylpenicillin (MIC ≥ 0.5), oxacillin (MIC ≥ 4), clindamycin (MIC = 4), and vancomycin (MIC = 2). A previous study reported that hVISA may not only be associated with persistent bacteremia and treatment failure but may also be a precursor of the vancomycin intermediate *S. aureus* phenotype [23]. In Saudi Arabia, the occurrence of community- and hospital-acquired methicillin-resistant *S. aureus* infections is recorded; however, there are no available reports regarding hVISA [24].

We found that *A. otitis*, a cause of acute otitis media, was resistant to clindamycin (MIC = 4), erythromycin (MIC ≥ 8), vancomycin (MIC = 1), nitrofurantoin (MIC = 128), and teicoplanin (MIC = 4). *A. otitidis* has been frequently documented as one of the most prevalent bacteria in middle ear aspirates of patients with otitis media with effusion [25]. Recently, it was reported that *A. otitidis* plays a role in the pathogenesis of otitis media with effusion, in which it forms both single- and multi-species biofilms with other bacteria, thus promoting multidrug resistance [26].

### 3.4. Questionnaires

**Figure 2:**
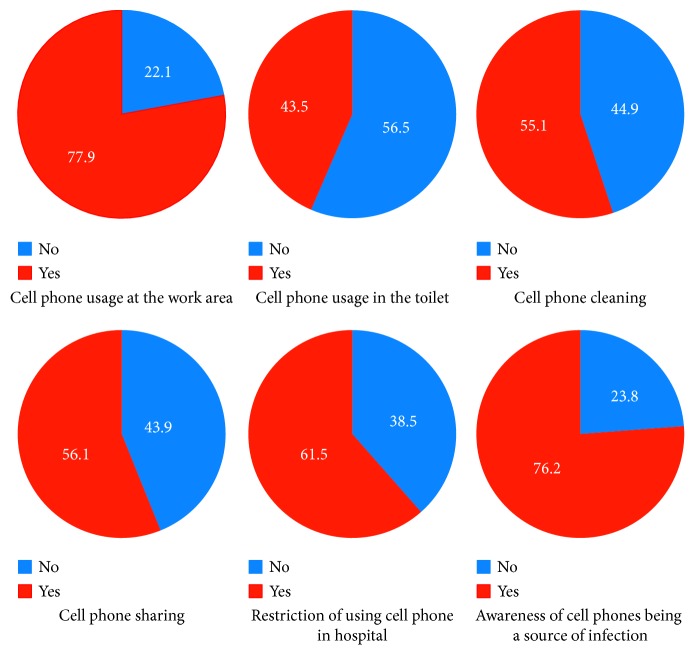
Survey results of cell phone use among healthcare workers (*n*=285) at selected hospitals.

Based on completed questionnaires, we found that 222 (77.9%) participants used their cell phones at work, 160 (56.1%) shared their phone with colleagues, and 128 (44.9%) never cleaned their phones. In addition, 23.8% of participants (68/285) believed that cell phones could serve as a source of bacterial transmission, and over half of the participants (61.5%) reported that they agreed with restriction rules for using cell phones in the college. However, according to the opinions of participants, 110 (38.5%) did not agree with these rules (Figure 2).

Data on the correlation between contamination level and questionnaire variables are shown in Table 3. There was a significant correlation between the contamination level and usage of cell phone in toilet and sharing (*P* <  0.05). By contrast, no significant correlation was found between contamination level and the usage of cell phones at the work area, cleaning cell phones by disinfectants, restriction of using cell phone at work, and awareness that cell phones can serve as a source of infection. There was, however, a positive correlation between the contamination level and the usage of cell phones at the work area and cleaning cell phones by disinfectants. Mkrtchyan et al. [27] reported that *Staphylococcus* species are common toilets isolates, and 37.8% of the isolates were drug resistant which can be freely transferred to the environment. Bhoonderowa et al. [28] reported that sharing mobile phone within females was associated with high bacterial load. It was recommended by previous studies that the level of bacterial contamination on the cell phones of healthcare workers can be reduced by reduce sharing [29].

## 4. Conclusion

Our study demonstrably highlights that the cell phones of healthcare workers can be contaminated by a wide range of bacteria including multidrug resistance bacteria. Bacteria may be readily able to adhere to the surface of cell phones, and the heat emitted by the cell phone enhances bacterial growth. These bacteria can then be transferred to the owner of the cell phone, patients, and the community. Based on our presented data, there is a lack of awareness of using cell phones in toilets and sharing among healthcare workers that may contribute to a significant risk of transmitting multidrug-resistant bacteria through unguarded cell phone use. The development of active preventive strategies is needed to reduce the risk of cross infection.

## Figures and Tables

**Table 1:** Types of bacteria isolated from cell phones of healthcare workers in selected hospitals.

Total	Hospital C	Hospital B	Hospital A	Bacterium
1	1	—	—	*Staphylococcus aureus*
9	2	—	7	*Staphylococcus hominis* subsp. *hominis*
10	1	2	7	*Staphylococcus epidermidis*
1	—	1	—	*Staphylococcus lentus*
1	—	—	1	*Staphylococcus capitis*
2	2	—	—	*Staphylococcus warneri*
3	—	2	1	*Micrococcus luteus*
1	—	—	1	*Globicatella sulfidifaciens*
1	—	—	1	*Facklamia hominis*
2	—	2	—	*Alloiococcus otitis*
2	—	2	—	*Dermacoccus nishinomiyaensis*
1	—	1	—	*Kocuria rosea*
1	—	1	—	*Alcaligenes faecalis* subsp. *faecalis*
1	—	1	—	*Vibrio fluvialis*
1	—	1	—	*Acinetobacter lwoffii*
1	—	1	—	*Pseudomonas stutzeri*
**38**	**6**	**14**	**18**	**Total**

**Table 2:** Antibiotic susceptibility against selected Gram-positive bacteria.

Antibiotic	*Staphylococcus aureus* (1)			*Staphylococcus hominis* (9)			*Staphylococcus epidermidis* (10)			*Staphylococcus lentus* (1)			*Staphylococcus warneri* (2)			*Alloiococcus otitis* (2)		
	S	I	R	S	I	**R**	S	I	R	S	I	R	S	I	**R**	S	I	R
Benzylpenicillin	0	0	**1**	1	0	**8**	0	0	**10**	1	0	0	0	0	**2**	NA	NA	NA
Oxacillin	0	0	**1**	7	0	**2**	7	0	**3**	0	0	**1**	0	0	**2**	NA	NA	NA
Gentamicin	1	0	0	9	0	**0**	10	0	0	0	0	**1**	2	0	0	NA	NA	NA
Tobramycin	0	0	**1**	8	0	**1**	7	1	**2**	1	0	0	2	0	0	NA	NA	NA
Erythromycin	0	0	**1**	3	0	**6**	5	0	**5**	0	1	0	1	0	**1**	0	0	**2**
Clindamycin	1	0	0	6	0	**3**	7	0	**3**	0	0	**1**	2	0	0	0	0	**2**
Linezolid	1	0	0	9	0	0	10	0	0	1	0	0	2	0	0	2	0	0
Teicoplanin	1	0	0	7	2	0	8	2	0	1	0	0	2	0	0	0	0	**2**
Vancomycin	0	1	0	9	0	0	10	0	0	1	0	0	2	0	0	0	0	**2**
Tetracycline	1	0	0	5	0	**4**	8	0	**2**	1	0	0	2	0	0	2	0	0
Fosfomycin	0	0	**1**	0	0	**9**	8	0	**2**	0	0	**1**	0	0	**2**	NA	NA	NA
Fusidic acid	0	1	0	1	1	**7**	0	5	**5**	0	1	0	1	0	**1**	NA	NA	NA
Rifampicin	0	1	0	0	9	0	0	10	0	0	1	0	0	2	0	NA	NA	NA
Trimethoprim/sulfamethoxazole	1	0	0	9	0	0	10	0	0	1	0	0	2	0	0	0	2	0

*Note.* I, intermediate; NA, not applicable; *R*, resistant; S, susceptible. Values in brackets indicate number of isolates. Number of resistant isolates indicated in bold.

**Table 3:** Correlation between contamination of cell phone and questionnaire variables.

Contamination	Correlation	*P* value
Cell phone usage at work area	0.897	0.290
Cell phone usage in toilet	0.992	0.038^*∗*^
Cell phone cleaning	0.830	0.375
Cell phone sharing	0.993	0.042^*∗*^
Restriction of using cell phone at work	−0.961	0.176
Awareness of being a source of infection	−0.847	0.356

^*∗*^Statistical analysis is significant, *P* < 0.05.

## Data Availability

The data used to support the findings of this study are available from the corresponding author upon request.

## References

[1] Famurewa O., David O. (2009). Cell phone: a medium of transmission of bacterial pathogens. *World Rural Observations*.

[2] Mahfouz A. A., Al Azraqi T. A., Abbag F. I., Al Gamal M. N., Seef S., Bello C. S. (2010). Nosocomial infections in a neonatal intensive care unit in south-western Saudi Arabia. *Eastern Mediterranean Health Journal*.

[3] Mark D., Leonard C., Breen H., Graydon R., O’Gorman C., Kirk S. (2014). Mobile phones in clinical practice: reducing the risk of bacterial contamination. *International Journal of Clinical Practice*.

[4] Ducel G., Fabry J., Nicolle L. (2002). *Prevention of Hospital Acquired Infections: A Practical Guide*.

[5] Shittu A., Oyedara O., Abegunrin F. (2012). Characterization of methicillin-susceptible and –resistant staphylococci in the clinical setting: a multicentre study in Nigeria. *BMC Infectious Diseases*.

[6] Akinyemi K. O., Atapu A. D., Adetona O. O., Coker A. O. (2009). The potential role of mobile phones in the spread of bacterial infections. *Journal of Infection in Developing Countries*.

[7] Tian Y., Zheng B., Wang B., Lin Y., Li M. (2016). Rapid identification and multiple susceptibility testing of pathogens from positive-culture sterile body fluids by a combined MALDI-TOF mass spectrometry and vitek susceptibility system. *Frontiers in Microbiology*.

[8] Cody W. L., Wilson J. W., Hendrixson D. R. (2008). Skim milk enhances the preservation of thawed −80°C bacterial stocks. *Journal of Microbiological Methods*.

[9] Lai C.-C., Chu C.-C., Cheng A., Huang Y.-T., Hsueh P.-R. (2015). Correlation between antimicrobial consumption and incidence of health-care-associated infections due to methicillin-resistant *Staphylococcus aureus* and vancomycin-resistant enterococci at a university hospital in Taiwan from 2000 to 2010. *Journal of Microbiology, Immunology and Infection*.

[10] Fleming K., Randle J. (2006). Toys-friend or foe? A study of infection risk in a paediatric intensive care. *Pediatric Nursing*.

[11] Karabay O., Koçoglu E., Tahtaci M. (2007). The role of mobile phones in the spread of bacteria associated with nosocomial infections. *Journal of Infection in Developing Countries*.

[12] Srikanth P., Ezhil R., Suchitra S., Anandhi I., Maheswari U., Kalyani J. The mobile phone in a tropical setting emerging threat for infection control.

[13] Zakai S., Mashat A., Abumohssin A. (2016). Bacterial contamination of cell phones of medical students at King Abdulaziz University, Jeddah, Saudi Arabia. *Journal of Microscopy and Ultrastructure*.

[14] Selim H. S., Abaza A. F. (2015). Microbial contamination of mobile phones in a healthcare setting in Alexandria, Egypt. *GMS Hygiene and Infection Control*.

[15] Otto M. (2009). Staphylococcus epidermidis—the ‘accidental’ pathogen. *Nature Reviews Microbiology*.

[16] Gatermann S. G., Koschinski T., Friedrich S. (2007). Distribution and expression of macrolide resistance genes in coagulase-negative staphylococci. *Clinical Microbiology and Infection*.

[17] Asaad A. M., Ansar Q. M., Mujeeb H. S. (2016). Clinical significance of coagulase-negative staphylococci isolates from nosocomial bloodstream infections. *Infectious Diseases*.

[18] Bouchami O., Achour W., Mekni M. A., Rolo J., Hassen A. B. (2011). Antibiotic resistance and molecular characterization of clinical isolates of methicillin-resistant coagulase-negative staphylococci isolated from bacteremic patients in oncohematology. *Folia Microbiologica*.

[19] Becker K., Heilmann C., Peters G. (2014). Coagulase-negative staphylococci. *Clinical Microbiology Reviews*.

[20] Harrison E. M., Paterson G. K., Holden M. T. G. (2013). A novel hybrid SCCmec-mecC region in *Staphylococcus sciuri*. *Journal of Antimicrobial Chemotherapy*.

[21] Szczuka E., Krzymińska S., Bogucka N., Kaznowski A. (2017). Multifactorial mechanisms of the pathogenesis of methicillin-resistant *Staphylococcus hominis* isolated from bloodstream infections. *Antonie van Leeuwenhoek*.

[22] Satola S. W., Farley M. M., Anderson K. F., Patel J. B. (2010). Comparison of detection methods for heteroresistant vancomycin-intermediate *Staphylococcus aureus*, with the population analysis profile method as the reference method. *Journal of Clinical Microbiology*.

[23] Casapao A. M., Leonard S. N., Davis S. L. (2013). Clinical outcomes in patients with heterogeneous vancomycin-intermediate *Staphylococcus aureus* bloodstream infection. *Antimicrobial Agents and Chemotherapy*.

[24] Abulreesh H. H., Organji S. R., Osman G. E. H., Elbanna K., Almalki M. H. K., Ahmad I. (2017). Prevalence of antibiotic resistance and virulence factors encoding genes in clinical *Staphylococcus aureus* isolates in Saudi Arabia. *Clinical Epidemiology and Global Health*.

[25] Chan C. L., Wabnitz D., Bassiouni A., Wormald P.-J., Vreugde S., Psaltis A. J. (2017). Identification of the bacterial reservoirs for the middle ear using phylogenic analysis. *JAMA Otolaryngology–Head & Neck Surgery*.

[26] Chan C., Richter K., Wormald P., Psaltis A., Vreugde S. (2017). *Alloiococcus otitidis* forms multispecies biofilm with *Haemophilus influenzae*: effects on antibiotic susceptibility and growth in adverse conditions. *Front Cell Infect Microbiol*.

[27] Mkrtchyan H. V., Russell C. A., Wang N., Cutler R. R. (2013). Could public restrooms Be an environment for bacterial resistomes?. *PLOS One*.

[28] Bhoonderowa A., Gookool S., Biranjia-Hurdoyal S. D. (2014). The importance of mobile phones in the possible transmission of bacterial infections in the community. *Journal of Community Health*.

[29] Dogan M., Feyzioglu B., Ozdemir M., Baysal B. (2008). Investigation of microbial colonization of computer keyboards used inside and outside hospital environments. *Mikrobiyoloji Bülteni*.

